# Unicellular versus Filamentous: The Glacial Alga *Ancylonema alaskana* comb. et stat. nov. and Its Ecophysiological Relatedness to *Ancylonema nordenskioeldii* (Zygnematophyceae, Streptophyta)

**DOI:** 10.3390/microorganisms9051103

**Published:** 2021-05-20

**Authors:** Lenka Procházková, Tomáš Řezanka, Linda Nedbalová, Daniel Remias

**Affiliations:** 1 Department of Ecology, Faculty of Science, Charles University, Viničná 7, 128 44 Prague, Czech Republic; lindane@natur.cuni.cz; 2 Institute of Microbiology, The Czech Academy of Sciences, Vídeňská 1083, 142 20 Prague, Czech Republic; rezanka@biomed.cas.cz; 3 Centre for Phycology, Institute of Botany of the Czech Academy of Sciences, Dukelská 135, 379 82 Třeboň, Czech Republic; 4 School of Engineering, University of Applied Sciences Upper Austria, Stelzhamerstr. 23, 4600 Wels, Austria

**Keywords:** cryoflora, supraglacial communities, photosynthesis, lipidome, fatty acids, polyphenols, Mesotaeniaceae, phylogeny

## Abstract

Melting polar and alpine ice surfaces frequently exhibit blooms of dark pigmented algae. These microbial extremophiles significantly reduce the surface albedo of glaciers, thus accelerating melt rates. However, the ecology, physiology and taxonomy of cryoflora are not yet fully understood. Here, a Swiss and an Austrian glacier dominated either by filamentous *Ancylonema nordenskioeldii* or unicellular *Mesotaenium berggrenii* var. *alaskanum*, were sampled. Molecular analysis showed that both species are closely related, sharing identical chloroplast morphologies (parietal-lobed for *Ancylonema* vs. axial plate-like for *Mesotaenium sensu stricto*), thus the unicellular species was renamed *Ancylonema alaskana*. Moreover, an ecophysiological comparison of the two species was performed: pulse–amplitude modulated (PAM) fluorometry confirmed that they have a high tolerance to elevated solar irradiation, the physiological light preferences reflected the conditions in the original habitat; nonetheless, *A. nordenskioeldii* was adapted to higher irradiances while the photosystems of *A. alaskana* were able to use efficiently low irradiances. Additionally, the main vacuolar polyphenol, which effectively shields the photosystems, was identical in both species. Also, about half of the cellular fatty acids were polyunsaturated, and the lipidome profiles dominated by triacylglycerols were very similar. The results indicate that *A. alaskana* is physiologically very similar and closely related but genetically distinct to *A. nordenskioeldii*.

## 1. Introduction

Melting glaciers harbor diverse communities of specialized microorganisms [1], and the habitat can be regarded as extreme for photoautotrophic life, considering the prevailing conditions [2,3]. Significant abiotic parameters are excessive irradiation, limited nutrients, permanently low temperature and diurnal freeze-thaw cycles on bare ice. The current knowledge about biodiversity, distribution and adaptations of glacial algae were reviewed in [4]. Generally, these habitats are dominated by few zygnematophycean species. Most common are *Mesotaenium berggrenii* var. *alaskanum* and *Ancylonema nordenskioeldii* (Zygnematophyceae, Streptophyta). Both species are closely related but morphologically distinguishable, the first being more roundish after cell cleavage and practically unicellular [5,6]; the latter cylindrical-elongate and forming filaments [7]. They are apparently cosmopolitan in polar and mid-latitude mountainous regions [8,9]. In addition, *Cylindrocystis* is a third zygnematophycean genus with members causing or participating in glacial blooms, and cells can be distinguished by cell size, shape and chloroplast morphology [10]. Cytologically, all of these species share abundant brownish phenols in vacuoles; the main constituent, a glycosylated purpurogallin derivative pigment, is believed to play a significant role for tolerating high visible and ultraviolet irradiance, thus preventing either photoinhibition or DNA mutation effects [11,12]. During summer, blooms of glacial algae, in combination with cryoconite and inorganic particles, reduce the albedo of glacier surfaces, consequently accelerating melting, even affecting scenarios of global sea-level rise in the long term [13,14,15,16]. This “dark ice” phenomenon is of extraordinary significance in the Western Greenland Ice Sheet [17].

The aim of this study was to explore similarities and differences between *M. berggrenii* var. *alaskanum* and *A. nordenskioeldii* from the European Alps. To this end, field populations dominated either by the first in Austria [6] or the later in Switzerland (recently described by [18]) were investigated. The taxonomic relatedness was evaluated by 18S rDNA and *rbc*L marker sequences, resulting in a retyping of the widespread unicellular species. This was complemented by comparing the light-dependent photophysiological performance and the cellular fatty acid and lipidomic profiles. Moreover, the phenolic pigment already characterized for *M. berggrenii* var. *alaskanum* [11] was analysed in *A. nordenskioeldii* as well. In general, we hypothesized on the one hand, that the physiology of both species should be similar. On the other hand, both morphotypes represent independent species, despite the fact that both algae commonly (but not necessarily) occur together in virtually all glaciated regions of the world.

## 2. Materials and Methods

### 2.1. Harvest and Field Measurements

Table 1 shows the collection details for *Mesotaenium berggrenii* var. *alaskanum* and *Ancylonema nordenskioeldii*. The presence and species composition of glacial algae were evaluated by an Evolution Portable Field Microscope (Ted Pella, Redding, CA, USA) at 400× magnification. Harvest took place with ice axes, ice saws and stainless-steel shovels by scrapping off the uppermost 1 to 2 cm of surface ice into 10 l buckets for transportation to the lab. The prevailing irradiance at the time of sampling was recorded for Morteratsch Glacier at an elevation of approximately 2300 m a.s.l. with PMA2100 logger (Solar Light Company, Glenside, PA, USA) using visible (VIS, PMA2131), ultraviolet-A (UVA, PMA2111) and UVB (PMA2106) sensors, placed 50 cm above the ice surface. pH and electrical conductivity were obtained with WTW instruments (Cond 340i Inolab, Weilheim, Germany).

### 2.2. Cell Cleaning, Storage and Microscopy

With a field microscope, virtually monospecific blooms (P39, WP211, WP167 and WP251) were harvested according to [19]. Additionally, three mixed algal snow communities (WP210, WP249, WP213) were included in this study to describe cell size ranges of the two studied glacial algal species. Harvested glacial ice was placed in 10 L bucket. The glacier ice was gently melted in the dark at 4 °C over night. In the first step, larger debris was removed by sieving the meltwater through 800, 400, 200 and 140 µm mesh size stainless steel sieve tower (Retsch, Germany). Samples WP211 and WP167 were subject of Sanger sequencing, pulse–amplitude modulated (PAM) fluorometric measurements and lipidomic analysis. Samples WP211 and WP251 were used for fatty acid analysis and cultivation assays.

To separate glacial algae from other microscopic objects like pollen, yeast, protozoa or other algae, a density gradient with Percoll (Merck P1644; layers of 50, 75 and 100% concentration, *v*/*v*) was performed and centrifuged (400× *g*, 10 min at 1 °C). The glacial algae accumulated at the 75% to 100% Percoll interface, were transferred with a syringe into a new centrifugation tube, and washed and centrifuged twice with sterile-filtered (Whatman GF/F) meltwater. Cleaned cells were stored at 2 to 4 °C prior cultivation assays at an illumination of approximately 40 µmol PAR m^−2^ s^−1^.

Cells were observed with a Nikon Eclipse 80i light microscope equipped with a Nikon DS-5M camera (Nikon Instruments, Amsterdam, Netherlands) for bright field and with a Zeiss Axiovert 200M for fluorescence. Cell concentrations were calculated based on counting with a Plankton Chamber acc. to Kolkwitz (Hydro-Bios, Altenholz, Germany).

### 2.3. Cultivation Assays

For obtaining strains of *A. nordenskioeldii* (WP211) and *M. berggenii* var. *alaskanum* (WP251), 10 µL aliquots of cells from glacial meltwater were inoculated on 1.7% (*w*/*v*) agar plates with synthetic freshwater medium (SFM, https://www.uni-due.de/biology/ccac/growth_media_sfm.php (accessed on 27 August 2020)) and incubated at approximately 5 µmol PAR m^−2^ s^−1^ irradiance at a diurnal regime of 12 h light/darkness at 4 °C.

### 2.4. Molecular Characterisation

DNA isolation out of the unialgal field samples WP211 and WP167 was carried out with a DNeasy Plant Mini Kit (Qiagen, Germany), as in [20]. The 18S small subunit ribosomal RNA gene (18S rDNA) and ribulose-1,5-bisphosphate carboxylase/oxygenase large subunit (*rbc*L) marker regions were amplified from DNA isolates by polymerase chain reaction (PCR) using existing primers (Table S1). Amplification reactions were described in [20]. PCR products were purified and sequenced using an Applied Biosystems automated sequencer (ABI 3730xl) at Macrogene Europe (Amsterdam, The Netherlands). The obtained sequences were submitted to NCBI Nucleotide sequence database (accession numbers of *Ancylonema nordenskioeldii* WP211: 18S rDNA—MW922838, *rbc*L—MW922839; *Ancylonema alaskana* WP167: *rbc*L—MW922840).

### 2.5. Phylogenetic Analysis

Two different alignments were constructed for the phylogenetic analyses, based on the 18S rRNA and *rbc*L gene sequences. The sequences were selected according to [10,11,21] to encompass all the zygnematophycean lineages. The 18S rDNA alignment contained 56 sequences (1777 bp), the *rbc*L matrix consisted of 49 sequences (1290 bp); Embryophytes (*Sphagnum palustre*, *Phaeoceros laevis*, *Marchantia polymorpha*) were selected as the outgroup. The best-fit nucleotide substitution model was estimated by jModeltest 2.0.1 [22]. Based on the Akaike information criterion, the TrN + I + G and GRT + I + G model was selected for 18S rDNA and *rbc*L, respectively. The 18S rDNA and *rbc*L phylogenetic trees were inferred by Bayesian inference (BI) and maximum likelihood (ML) according to [23], with the minor modification that Markov Chain Monte Carlo runs were carried out for three million generations in BI. Convergence of the two cold chains was checked by the average standard deviation of split frequencies of 0.001262 and 0.001563. Bootstrap analyses and Bayesian posterior probabilities were performed as described by [23].

### 2.6. Photosynthesis

The light-dependent performance of photosystem II was measured with the unialgal field samples WP167 and WP211 (for sample processing see the Section 2.2). To gain sufficient biomass, cells were concentrated by passive sedimentation of melt water in a 1 L plastic cylinder at low light conditions at 4 °C overnight and the pellet was then used for measurements. Prior measurement, algae were kept in the glacial meltwater in the dark for 30 min. In vivo chlorophyll fluorescence parameters were obtained with a pulse–amplitude modulated fluorometer (PAM 2500, HeinzWalz GmbH, Pfullingen, Germany) in a 0.6 mL chamber thermostated to 1 °C. To obtain the relative electron transport rates (rETR), the apparent quantum yield for electron transport (alpha) and the light saturation point (I_k_), cells were exposed to photon flux densities (PFD) of 5, 9, 34, 67, 104, 201, 366, 622, 984, 1389 µmol photons m^−2^ s^−1^ for 30 s each. Four independent replicates were measured. For further technical details, see [20].

### 2.7. Analysis of Phenols

The phenolic compounds of *A. nordenskioeldii* were extracted and analysed according to [11]. Briefly, 20% ethanol extracts were characterized by liquid chromatography mass spectrometry (LC-MS) with an Agilent ChemStation 1100, equipped with a Synergy Hydro column 4 µm column (Phenomenex) and a diode array detector set at 280 nm, coupled with an Esquire 3000plus mass spectrometer with electrospray ionization (Bruker Daltonics, Bremen, Germany).

### 2.8. Isolation and Fractionation of Lipids

The extraction procedure was based on the method of [24], with modification as previously described [25]. Briefly, the lyophilized cells were suspended in a dichloromethane-methanol mixture (2:1, *v*/*v*) for 30 min with stirring, after which dichloromethane and water were added and the dichloromethane phase was evaporated to dryness under reduced pressure. Total lipids were dissolved in the mobile phase (acetonitrile: 2-propanol 99:1, *v*/*v*)) for further analysis by shotgun lipidomics. Lipid classes were eluted from a Sep-Pak Vac Silica cartridge 35cc (Waters; 10 g normal-phase silica) by chloroform (neutral lipids), acetone (glycolipids), and methanol (phospholipids) according to [26]. All classes of lipids were saponified overnight in 10% KOH in methanol at room temperature. Fatty acid methyl esters were prepared by reaction of free fatty acids with diazomethane according to [20].

### 2.9. Shotgun Lipidomics

An LTQ-Orbitrap Velos mass spectrometer (Thermo Fisher Scientific, San Jose, CA, USA), a high-resolution hybrid mass spectrometer equipped with a heated electrospray interface (H-ESI), was operated in positive and negative ionization mode. Flow injection analysis (FIA) was used for sample introduction into the H-ESI ion source. Acetonitrile/water (50:50, *v*/*v*) was used at the flow rate of 150 μL/min. The MS scan range was performed in the Fourier transform mode and recorded within a window between 200–2000 *m*/*z*. Mass spectra were acquired with target mass resolution of R = 110,000 at *m*/*z* 800 and the ion spray voltage was set at +3.5 kV (in the positive ionization mode) and –2.5 kV (in the negative ionization mode). Both ionization modes used the following parameters: sheath gas flow, 18 arbitrary units (AU); auxiliary gas flow, 7 AU; ion source temperature, 250 °C; capillary temperature, 230 °C; capillary voltage, 50 V; and tube lens voltage, 170 V. Helium was used as a collision gas for collision-induced dissociation (CID) experiments. The CID normalization energy of 35% was used for the fragmentation of parent ions. The MS/MS product ions were detected by the high resolution FT mode. The calibration of the MS spectrometer was undertaken with the use of a Pierce LTQ Orbitrap positive and/or negative ion calibration solution (Thermo Fisher Scientific, San Jose, CA, USA). The internal lock mass was used in mass spectra acquisition, i.e., 255.2330 *m*/*z* [M-H]^−^ palmitic acid in the negative ESI. The mass accuracy was better than 0.9 ppm. The chemical structure of the compounds was confirmed with the help of the spectral database LIPID MAPS^®^ Lipidomics Gateway (http://www.lipidmaps.org/ (accessed on 15 December 2020)).

### 2.10. Fatty Acid Methyl Esters Analysis (FAMEs)

The structures of fatty acid methyl esters (FAMEs) were confirmed by comparison with gas chromatography/mass spectrometry retention times and fragmentation patterns with those of standard FAMEs (Supelco, Prague, Czech Republic) [27]. Procedures were described in detail in [20].

## 3. Results

### 3.1. Field Blooms

Microalgal ice surface blooms at Morteratsch Glacier in Switzerland were mainly caused by filaments of *A. nordenskioeldii* and some scattered unicells of *M. berggrenii* var. *alaskanum*. In contrast, the blooms at Gurgler Ferner in Austria, which were situated approximately 90 km northeast from the Swiss site, consisted microscopically exclusively of *M. berggrenii* var. *alaskanum.* Based on the acquired morphological and genetic data from both field sites, the latter was moved to the genus *Ancylonema* and the rank of a variety is raised to species status (see below).

### 3.2. Taxonomic Treatment

**Figure 1:**
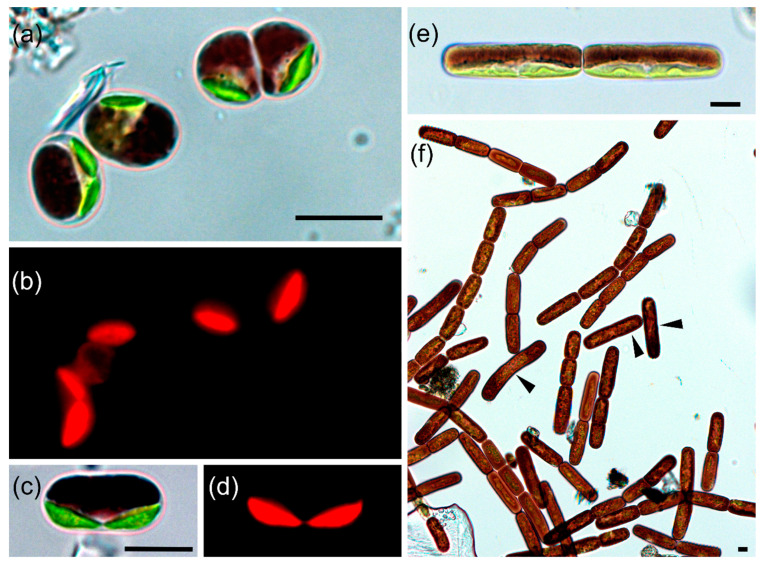
Light microphotographs of cells collected from melting glacier surfaces in the European Alps, (**a**–**d**) *Ancylonema alaskana* comb. et stat. nov. (WP167) and (**e**,**f**) *Ancylonema nordenskioeldii* (WP211). (**a**) transient stage of two cells after cleavage (upper right), showing one parietal chloroplast per cell in the side view (upper right and middle) or two chloroplasts before cell cleavage (lower left) (WP167), (**c**) elongated cell with two chloroplasts, (**a**–**d**) corresponding bright field and fluorescence images, the latter reveal the partly lobed chloroplast shape. (**e**) side view demonstrating two parietal chloroplasts per cell (or one with two pyrenoids), (**f**) group of dark pigmented filaments, most of them with 2, 4 or 8 cells, but also single individuals were present (arrowheads). Scale: 10 μm.

***Ancylonema alaskana* (Kol) Procházková and Remias comb. et stat. nov. (Figure 1a–d)**

BASIONYM: *Mesotaenium berggrenii* var. *alaskanum* Kol 1942, Smithsonian Miscellaneous Collections 101: pp. 25–26 (as “*alaskana*”)

EPITYPE (here designated): The field vegetative cells (specimen WP167) deposited in a lyophilized (non-viable) state at the Culture Collection of Algae of Charles University in Prague, Czech Republic. Figure 1a show the morphology of the cells within the specimen WP167 used for the epitype.

HABITAT: polar and alpine glacial ice surfaces, occasionally in melting snow and slush.

DISTRIBUTION (based on light microscopical diagnosis): Alaska [28,29,30], European Alps [6,11], Svalbard [31], Greenland [17,32], Altai Mts, Russia [33], Himalaya [34], Antarctica [5].

SPECIES DESCRIPTION: Cells single or in temporary connected pairs after cell division, cell walls smooth, cells 7–11.6 µm wide and 8.1–20.6 µm long, cylindrical cell shape with apexes broadly rounded, one (or two) parietal, discoid, cup-shaped or elongate-hemispherical, weakly lobed chloroplast with one pyrenoid, sometimes causing a bulge. Cytosol with central unpigmented nucleus and peripheral vacuoles, the latter occupied by characteristic dark phenols. Chloroplast division precedes cytokinesis. Sexual reproduction by conjugation between two cells results in a regular or irregular, spherical or angular to ellipsoidal zygote. DNA sequences available: nuclear 18S rDNA (JF430424) and plastid *rbc*L (MW922840).

FIELD SAMPLES EXAMINED: WP167, WP251.

REMARKS: *A. nordenskioeldii* occurs in the same habitat as *A. alaskana* and has the same brownish vacuolar pigmentation, but differs by significantly larger cell widths and cell lengths (10.7–15.2 µm × 19.6–51 µm; based on Swiss populations; this study), trichal habitus (filaments usually with 2 to 64 cells [7] and an irregularry oblong zygote [7]. Single cells of *A. nordenskioeldii* can be distuingished from *A. alaskana* in most cases by cells‘ size, the former reaching up to 15 × 65 µm (Greenland, figures 10, 11 in [35] or 15 × 32–72 µm (Swiss Alps, Figure 1f, this study). However, singe cells of *A. nordenskioeldii* can be morphologically confused with individuals of „large“ *M. berggrenii sensu* [8], a taxon with an unclear status (no molecular sequence available yet). Finally, *A. nordenskioeldii* and *A. alaskana* differ in their 18S rDNA and *rbc*L molecular markers. For *A. nordenskioeldii* from Morteratsch Glacier, accession numbers are provided: 18S rDNA—MW922838; *rbc*L—MW922839.

NOMENCLATURAL REMARKS: *Ancylonema nordenskioeldii* var. *berggrenii* Wittrock (Tafel III, figure 18) [36] (≡ *Mesotaenium berggrenii* (Wittrock) Lagerheim [37]) is a valid infraspecific name based on the articles 38.8–38.10 of the International Code of Nomenclature for algae, fungi, and plants [38]. *M. berggrenii* var. *alaskanum* should be treated as a distinct species from its type variety based on the cell morphological differences. For unambiguous nomenclatural identification, the proposed comb. nov. was based on the variety epithet of “*alaskanum*” from the basionym (reflecting the initial description from Alaska by [28]), which is here promoted as the species epithet “*alaskana*”.

### 3.3. Habitat Conditions, Population Densities and Cell Sizes of Glacial Algae

The glacier communities and the habitat parameters between the Austrian and the Swiss site were compared. At Gurgler Ferner (Austria), the sampling sites were at the lower, snow-free and flat parts of ice where greyish to purple surface blooms were observed during August 2017 and 2020 (Figure S1a), with cells of *A. alaskana* (Figure 1a–d). The meltwater pH was 5.3 and the EC 4.3 µS cm^−1^. At Morteratsch Glacier (Switzerland), a similar situation was found in August 2018 and 2020 (Figure S1b) and was dominated by *A. nordenskioeldii* (Figure 1e,f), comprising usually of 2 to 8 cells per filament (longest observed filament was 16 cells), and the meltwater had a pH of 6.1 and an EC of 4.4 µS cm^−1^. At 12:50 h Central European Time, the following irradiance values were recorded at Morteratsch Glacier (during sunshine): photosynthetically active radiation (PAR): 2113 µmol photons m^−2^ s^−1^, UV-A: 4.55 mW cm^−2^ and UV-B: 0.158 W m^−2^. While *A. alaskana* was present at both glaciers, filaments of *A. nordenskioeldii* were not found at Gurgler Ferner. Additionally, a few filaments morphologically addressable as *Ancylonema nordenskoeldii* var. *chodatii* were present at Morteratsch Glacier (Figure S2). In all cases, the cytosol was abundantly occupied by brownish pigmented vacuoles. Abundances and cell sizes are summarized in Table 2. The maximum population densities were quite similar on both glaciers. Generally, the cell width showed less variations, while cell lengths showed more variation due to prolonged growth prior cell division. Cells of *A. alaskana* had significantly less width (Mann–Whitney test, *p* < 0.0001) and significantly less length (Mann–Whitney test, *p* < 0.0001) compared to *A. nordenskioeldii* (Figure S3).

### 3.4. Cultivation Attempts

Under laboratory conditions, the field collected cells transiently continued for several weeks to grow (including a few cell divisions), the dark vacuolar pigmentation decreased and chloroplast morphology became evident (Figure S4). In the case of *A. nordenskioeldii*, it resulted in the formation of filaments with up to 64 cells (data not shown). Similarly, *A. alaskana* developed single cells, short and loose filaments with up to 4 cells. No strain was established.

### 3.5. Molecular Taxonomy

The population of *A. nordenskioeldii* from the Swiss Alps was 100% identical with Svalbard ones for the 18S rDNA molecular marker, i.e., compare MW922838 and AF514397.2. Due to the longer 18S rDNA fragment of a newly acquired sequence (2008 bp), the intron close to the 3’-end of 18S rDNA sequence of this species was detected only for the former (accession number MW922838), corresponding by its position to intron group I in *Fottea pyrenoidosa* SAG 1.88 (1760-2063 in KM020068.1), while the available sequences of polar *A. nordenskioeldii* (1753 bp long, AF514397.2) and alpine *A. alaskana* were shorter (1660 bp long, JF430424.1).

**Figure 2:**
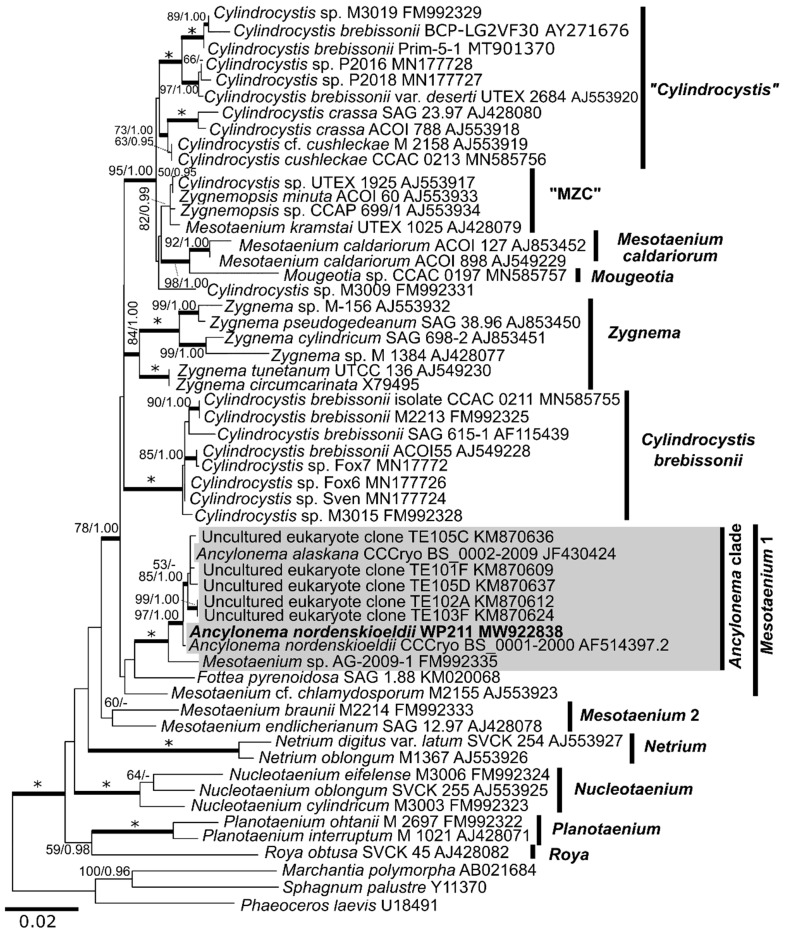
18S rRNA gene-based maximum-likelihood phylogenetic tree of the Zygnematophyceae. The ‘*Ancylonema*’-clade is highlighted (grey box). All the other labelled clades correspond to [21]. Posterior probabilities (≥0.95) and bootstrap values from maximum likelihood analyses (≥50%) are shown. Full statistical support (1.00/100) is marked with an asterisk. Thick branches represent nodes receiving the highest posterior probability support (1.00). Newly obtained sequence is in bold. Accession numbers, strain or field sample codes are indicated after each species name.

**Figure 3:**
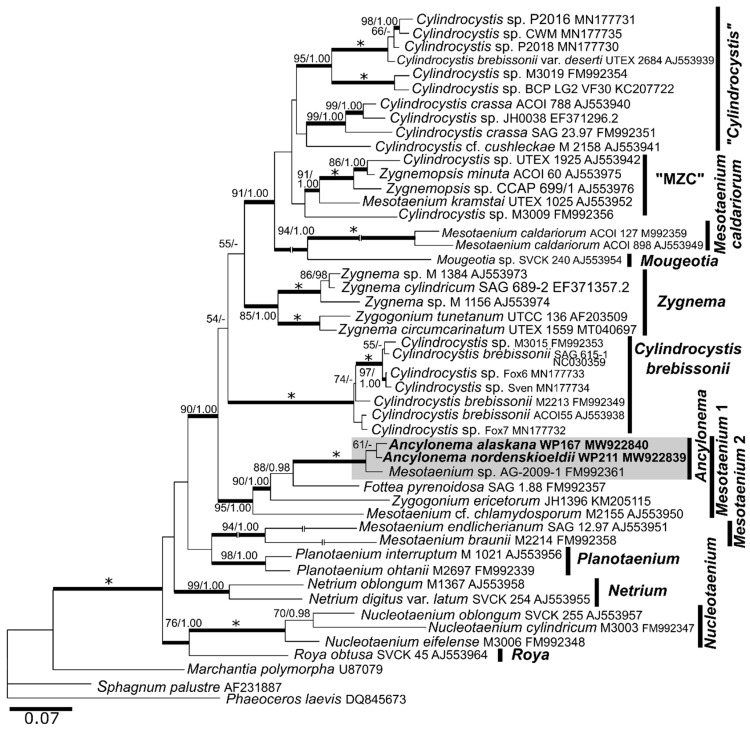
The *rbc*L gene-based maximum-likelihood phylogenetic tree of selected Zygnematales. The names of clades correspond to [21]. Posterior probabilities (≥0.95) and bootstrap values from maximum likelihood analyses (≥50%) are shown. Full statistical support (1.00/100) is marked with an asterisk. Thick branches represent nodes receiving the highest posterior probability support (1.00). Newly obtained sequence in bold. Accession numbers, strain or field sample codes are indicated after each species name.

The phylogenetic analysis of 18S rDNA within the Zygnematales showed that the two investigated glacial algae were very closely related (Figure 2). They formed an independent, well-supported “*Ancylonema*“ clade. The sequence difference in 18S rDNA marker was between the two species 3 out of 1552 bp (99.8% similarity; MW922838 vs. JF430424.1). According to the *rbc*L phylogeny, *A. nordenskioeldii* and *A. alaskana* congruently formed a well-supported clade with *Mesotaenium* sp. AG-2009-1, which was resolved as a part of a larger clade “*Mesotaenium* 1” (Figure 3). Further members of the *Mesotanium* 1-clade were *Fottea pyrenoidosa* SAG 1.88 (from soil in subantarctic Signy Island), *Zygogonium ericetorum* JH1396 (Austrian Alps) and *Mesotaenium* cf. *chlamydosporum* M-2155 (unknown origin). The sequence difference in the *rbc*L marker between the two glacial algae was 6 out of 558 bp (98.9% similarity; MW922839 vs. MW922840).

### 3.6. Photosynthesis

**Figure 4:**
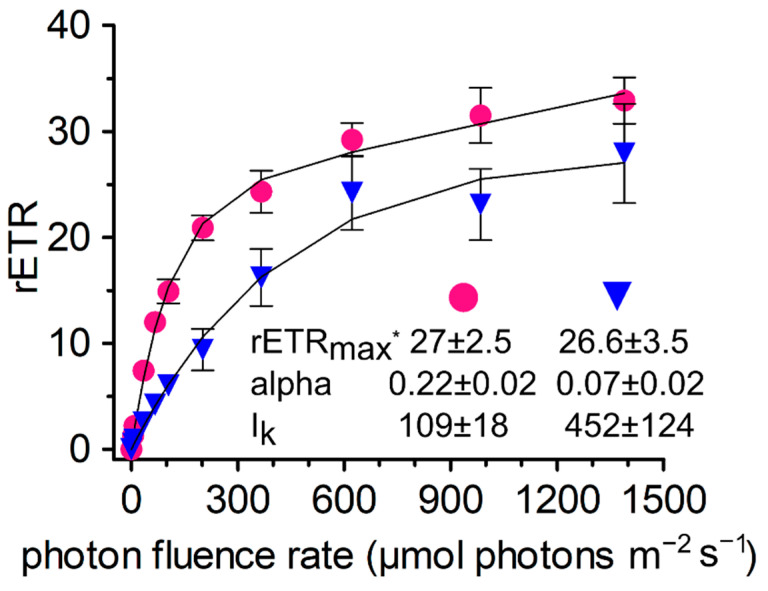
Photosynthetic rapid light curves of *Ancylonema alaskana* (pink circles) and *Ancylonema nordenskioeldii* (blue triangles). The effect of increasing photon fluence rates (*x*-axis) on the relative electron transport rate (rETR; *y*-axis) in chloroplasts was measured with field samples (WP167 and WP211) (n = 4, ±SD). The maximal rETR value achieved at 1500 µmol photons m^−2^ s^−1^ (*). The data points were fitted to the model, assuming no photoinhibition [39].

To compare the photophysiology of *A. alaskana* and *A. nordenskioeldii*, rapid light curves were generated (Figure 4). The maximal rETR achieved at 1500 µmol photons m^−2^ s^−1^ was 27 and 26.6, respectively, although a saturation of the light curves was not evident by this highest applied irradiance. The two glacial algae significantly differed in the values alpha (0.22 vs. 0.07) and I_k_ (109 vs. 452). Whilst higher alpha and lower I_k_ of *A. alaskana* indicated that its photosystem II is working very effectively at very low irradiations, it remained capable of greater electron transport for a given PAR over the whole measurement range as compared to *A. nordenskioeldii*.

### 3.7. Phenolic Pigments

The main chromatographic peak of the aqueous extract of *A. nordenskioeldii* had online absorption maxima at 305 and 395 nm (Figure S5). Mass spectrometry of this compound revealed a molecular weight of 426 g mol^−1^.

### 3.8. Shotgun Lipidomic

**Figure 5:**
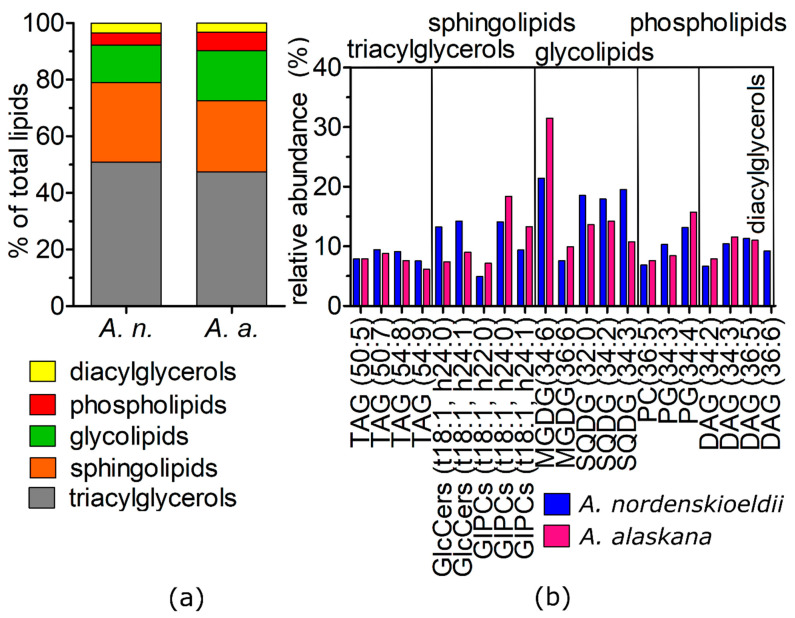
Cellular lipid composition of *Ancylonema nordenskioeldii* (WP211) and *Ancylonema alaskana* (WP167). (**a**) The relative proportions of lipid classes in (%) of total lipids. (**b**) The relative abundance of the most abundant lipid representatives of triacylglycerols (TAGs), sphingolipids (GIPCs, GlcCers), glycolipids (MGDG, SQDG), phospholipids (PG, PC),diacylglycerols (DAGs). The figure (**b**) shows only lipids that had abundances greater than 7% in a relevant lipid class.

Lipidomic profiles of the two *Ancylonema* species are shown in Figure 5 and described in detail in Table S2. Altogether, 346 molecular species of lipids and 19 lipid subclasses were identified. Triacylglycerols (TAGs) represented the most abundant lipid class of the *A. nordenskioeldii* and *A. alaskana* (50.9% vs. 47.4% in total lipids). These were followed by sphingolipids (28% vs. 25.2%; five subclasses), glycolipids (13.3% vs. 17.7%; three subclasses), phospholipids (4.3% vs. 6.6%; nine subclasses) and diacylglycerols (3.5% vs. 3.1%). Triacylglycerols of the two samples consisted only of molecules with an even number of acyl carbons, i.e., from 42 to 60. The most frequent were TAGs having 3–9 double bonds. The most abundant TAGs were 50:7, 54:8, 50:5 and 54:9. The most abundant sphingolipids were glycosyl inositol phospho ceramides (GIPCs) with sphingoid bases: t18:1, h24:0 and t18:1, h24:1, followed by glucosylceramide (GlcCers) (t18:1, h24:0 and t18:1, h24:1, i.e., sphingoid bases). The major glycolipids were monogalactosyldiacylglycerol (MGDG) 34:6 and sulfoquinovosyldiacylglycerol (SQDG) 32:0, 34:2, 34:3. The main phospholipids were phosphatidylglycerol (PG) 34:4 and phosphatidylcholine (PC) 36:5. Only small differences in the abundance of TAGs and the main representatives of the other lipid classes were detected between these algal species.

### 3.9. Fatty Acid (FA) Composition

**Figure 6:**
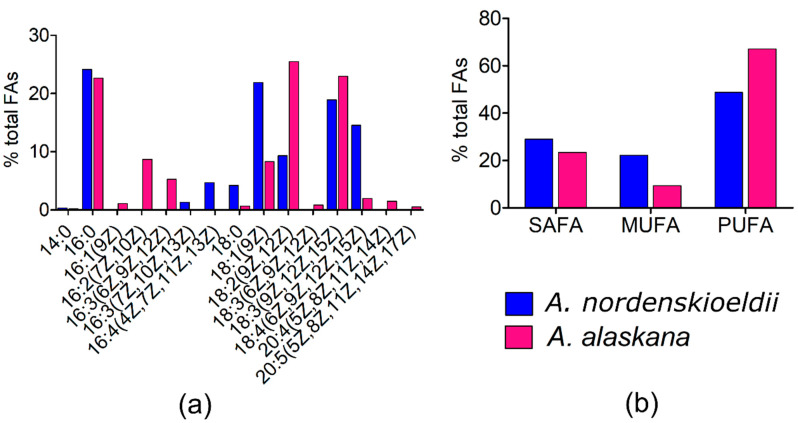
Cellular fatty acid composition *Ancylonema nordenskioeldii* (blue; WP211) and *Ancylonema alaskana* (pink; WP251). (**a**) The relative proportions of fatty acids in (%) of total fatty acids. **(b**) The relative proportion of saturated (SAFA), monounsaturated (MUFA), and polyunsaturated (PUFA) fatty acids. The figure (**a**) shows only fatty acids that had abundances greater than 0.1% of total FAs.

The relative content of FAs (in percentage of total fatty acids) in *A. nordenskioeldii* (WP211) and *A. alaskana* (WP251) is shown in Figure 6. FAs from 14 to 20 C were found. Cells accumulated high levels of PUFAs (48.8% and 67.1% of total lipids, respectively), whereas the content of saturated acids (SAFAs) did not exceed 29% and 23.8%, respectively (mainly palmitic acid (16:0), i.e., 24.1% and 22.6%, respectively). The main monounsaturated fatty acid (MUFA) was oleic acid (18:1 (9Z), 21.9% and 8.3%). The most abundant PUFAs were linoleic acid (18:2 (9Z, 12Z), 9.3% and 25.5%), α-linolenic acid (18:3 (9Z,12Z,15Z), 18.9% and 22.93%), followed by steriadonic acid (18:4 (6Z, 9Z, 12Z, 15Z), 14.5% and 2%), and hexadecatetraenoic acid (16:4 (4Z, 7Z, 11Z, 13Z), 4.7% and 0 %). The total lipid content per dry mass of *A. nordenskioeldii* and *A. alaskana* was 10.1% and 12.8%, respectively.

## 4. Discussion

### 4.1. Geographical Distribution of Glacial Algae

While *A. alaskana* seems to be widespread on many glaciers of the Eastern Alps (localities summarized in Table S3) and elsewhere, *A. nordenskioeldii* was hardly reported from Europe, indicating that it could require certain abiotic conditions to cause blooms. The latter species was initially described from the Greenland Ice Cap [35], where it grows abundantly during summer [16]. Reports of *Ancylonema* filaments from the southern hemisphere are rare: Southern Patagonia Icefield, Chile [40] and King George Island, Maritime Antarctica [41]. In Central Europe, the first discovery was by Chodat in 1896 at Mont Blanc, France [42]. Recently, a large population was discovered at Morteratsch Glacier, Switzerland [18] and subsequently investigated in this study. This glacier is one of the largest in the Eastern Alps, reaching down to altitudes of about 2.200 m a.s.l., where a prolonged melting period without snow cover on the ice probably supports *A. nordenskiöldii* in developing its characteristically trichal stages. Nonetheless, it is possible that this species is more widespread than expected, taking various reports of “large” *M. berggrenii* cells into account, which could represent single celled stages of *A. nordenskioeldii.*

The cellular characteristics of *A. alaskana* were similar to those reported previously from polar or high alpine regions such as Antarctica (“small form” in [5]), Himalaya [34], Svalbard [31], Greenland [17,32] and Alaska [28]. However, it remains currently unresolved whether *A. alaskana* is a single species with cosmopolitan distribution, or a number of similar but distinct unicellular taxa evolved growing on melting ice surfaces.

Also, in the case of *A. nordenskioeldii* the question of one species with global distribution remains under debate. Despite the lack of strains and marker sequences from field populations of many regions in the world, significant variation in average cell sizes between communities point either to an underestimated biological diversity of zygnematophycean glacial algae or certain levels of ploidy, as suggested by [5]. Moreover, single-celled stages of *A. nordenskioeldii* (Figure 1f), although already mentioned in the original description [35], may have been morphologically misinterpreted with different species, such as a large form of “*M. berggrenii*” *sensu* [8] (e.g., see figure 2C in [13]; and figure 1e in [18]). At Morteratsch Glacier, we noticed scattered filaments of an apparently third species, similar to the description of *A. nordenskioeldii* var. *chodatii* [43]. However, sequences of this organism are currently not available, but the distinct morphology indicates that these streptophytic organisms form characteristic size classes.

### 4.2. Habitat Conditions, Population Densities and Cell Sizes of Glacial Algae

The irradiation levels, slightly acidic pH and very low meltwater conductivity at the Swiss glacier were comparable to those at the studied Austrian glacier [6]. In both cases, the populations were sampled close to the glacier terminus, with the Austrian sampling site located 500 m higher. The population densities of glacial algae at the Austrian and Swiss sites reached values known from polar regions (up to 3 × 10^5^ cells mL^−1^, SW Greenland Ice Cap, [13]; 1 × 10^5^ cells mL^−1^ Windmill island, Antarctica, [5]) or were higher than these (up to 1.6 × 10^4^ cells mL^−1^, SE Greenland Ice Cap, [17]). However, these numbers were observations at a given moment in course of the season; episodic variations of physical parameters like precipitation and surface temperature influence the population development and consequently the algal biomass (T. Kohler, Charles University, Czech Republic—pers. observation), as indicated also by ice-core analysis [44]. Furthermore, the extent of blooms is influenced by inorganic nutrient availability in these oligotrophic habitats, which was demonstrated by [31] or [16].

Overall cell sizes were demonstrated here as a significant key in European localities to distinguish *A. alaskana* from *A. nordenskioeldii*. Interestingly, generally smaller *A. nordenskioeldii* were reported from Arctic Svalbard (8.1 ± 1.7 µm wide · 19.0 ± 7.8 µm long, [11]), these cells were genetically identical for 18S rDNA with the Swiss populations. It remains open as to whether the former represents a different, very closely related species, or their smaller sizes are an effect of different abiotic conditions than in the Alps.

### 4.3. Phylogeny and Morphological Traits

Molecular mechanisms during terrestrialisation of the Zygnematophyceae were highlighted by [45], namely that horizontally acquired genes from soil bacteria regulate cell development and stress response, like in their sister group, the land plants. This could have been a prerequisite for the colonization of wet ice surfaces as well. Nonetheless, the closest non-cryoflora relatives of glacial algae have not been elucidated yet; the majority of Mesotaeniaceae live in semi-terrestrial or aerophytic habitats. The 18S rDNA and *rbc*L phylogenies showed a very close relationship between *A. nordenskioeldii* and *A. alaskana*, pointing to a common ancestor, and they formed the *Ancylonema* clade. In contrast, the third prominent species of glacial algae, *Cylindrocystis brebissonii*, is situated in its own zygnematophycean clade which was named after this organism [10].

*A. alaskana* from Austria was very closely related to four unaffiliated environmental sequences isolated from an Alaskan glacier. Appropriately, this species (respectively its basionym) was initially described from Alaska [28]. The *rbc*L sequences obtained in this study confirmed the placement of the genus *Ancylonema* in the larger “*Mesotaenium* 1”-clade *sensu* [21]. The closest known, non-glacial 18S rDNA and *rbc*L sequences belonged to semi-terrestrial *Mesotaenium* AG-2009-1 isolated from wet rocks in a forest at Eifel, Germany (no strain available; A. Gontscharov, FEB RAS, Vladivostok, Russia—pers. comm.). It was placed into the *Ancylonema* clade, indicating that this clade is not exclusively glacial algae. The further closest relatives are the high alpine semi-terrestrial alga *Zygogonium ericetorum* from an intermittent streamlet in the Austrian Alps [46] and *Fottea pyrenoidosa* SAG 1.88, isolated from soil at subantarctic Signy Island [47]. While the latter had green cells, *Zygogonium* exhibited striking purple vacuoles [48] caused by phenols in a similar manner to glacial algae.

This study confirms, in accordance with earlier work (e.g., [21]), the polyphyly of *Mesotaenium*. Traditionally, the chloroplast morphology was crucial to distinguish between genera within the Zygnematales. *Mesotaenium* was typified by a single, central (axial) plate-like chloroplast (and prior cell division there are two of them) [49]. In contrast, the chloroplast of both *Ancylonema* species is clearly parietal, from discoid through slightly cup-shaped to flattened-lobed. *A. nordenskioeldii* was initially described as possessing a single chloroplast per cell [35]. However, the fact that two chloroplasts per cell were observed in this species as well [11] may either suggest that two independent plastids touch each other, or one plastid is almost divided in two parts, held together by a narrow sinus. The latter was described as “Charles bridge” for *Cylindrocystis* spp. [10]. Such a sinus has not been observed in field material of *A. alaskana* nor *A. nordenskioeldii* yet, but its presence cannot be excluded.

### 4.4. Photosynthesis and Phenols

Fluorometric photophysiology showed no decline of activity at higher irradiation in *A. nordenskioeldii* from Switzerland. Using different protocols, no drop of the light-dependent oxygen evolution took place in Arctic populations of filamentous *Ancylonema*, even at high VIS irradiation of 2000 µmol photons m^−2^ s^−1^ and more [7,12]. Similarly, *A. alaskana* from Austria performed well up to approximately 1400 µmol PAR m^−2^ s^−1^ (PreSens optical sensor, [11]). In this study, the two glacial algae differed in their photophysiological behavior at low light, *A. nordenskioeldii* was less effective (lower alpha), compared to *A. alaskana*. The low alpha and high I_k_ of *A. nordenskioeldii* from Switzerland is in agreement with values from a community at the Greenland Ice Sheet [17]. Here, a snapshot of the photophysiology of the two glacial algae was presented, close to the end of the summer season at the two different high Alpine sites (the Gurgler Ferner site for *A. alaskana* and the Morteratsch Glacier site for A. *nordenskioeldii*), and further measurements should reveal the variability within and between the species.

Glacial algae appear dark due to the massive accumulation of brownish aqueous phenols [11], and they can account almost 11 times the cellular content of chlorophyll-a [12]. These phenolic pigments, and probably further not yet elucidated components, serve as “sun screen” to protect the low-light adapted chloroplasts (I_k_ ∼46 μmol photons m^−2^ s^−1^; [12]. The photoprotective role of phenolic compounds is not exclusive to glacial algae, and is acknowledged also for non-cryoflora members of Zygnematales [50].

The main chromatographic peaks of *A. nordenskioeldii* and *A. alaskana* were identical by means of retention time, spectral absorption, molecular weight and MS fragmentation pattern [11]. It remains open whether purpurogallin carboxylic acid-6-O-β-D-glucopyranoside is also prevalent in other zygnematophycean algae with brownish vacuolar pigmentation, i.e., *Cylindrocystis brebissonii* [51] or *Temnogametum iztacalense* [52].

### 4.5. Lipidomics and Fatty Acids

Regarding the proportion of the lipid classes and relative proportions of lipid species, a very similar lipidome was found for *A. nordenskioeldii* and *A. alaskana*, reflecting that these species live in the same type of habitat. Here, a snapshot of lipid profiles of natural populations is presented which may be utilized as a reference for future laboratorial strain studies. One of the major thylakoid membrane lipids in algal chloroplasts are glycolipids, namely MGDG, DGDG and SQDG, and phospholipid PG [53]. Microalgae alter their MGDG to DGDG ratio in response to plenty of environmental factors [54]. The MGDG to DGDG ratio for *A. alaskana* and *A.* *nordenskioeldii* was 5.81 and 5.95, respectively. In general, a ratio of more than 2 is typical for microalgae grown under balanced growth [54], while the ratio decreases under conditions that are not favorable for growth (such as nutrient starvation, high light intensities) [54,55]. Membrane lipid changes play both structural and regulatory roles in plant adaptation and survival when exposed to temperature changes. Under the cold acclimation of a plant, phospholipids PC, glycolipids MGDG and DGDG species that contain two polyunsaturated acyl species, such as 36:5- (18:3–18:2) PC, 34:6- (18:3–16:3) MGDG, and 36:6-DGDG, increased [56]. In the studied glacial algae, these lipid species were the most abundant in their relevant lipid classes. Neutral lipids are represented by tri- and diacylglycerols, and in microalgae are usually deposited in lipid bodies [57]. The most abundant TAGs found in this study (50:7, 54:8, 54:9) were consistent with the most abundant TAGs found in other cryoflora, namely vegetative stages of in situ snow algal populations [58]. Additionally, sphingolipids are crucial elements in membrane organization where they are supposed to play a major role in raft formation and cross bi-layer lipids integration [59]. These compounds represented here the second most abundant lipid class. The desaturation of sphingolipids may be of essential importance in cold stress responses [60]. Namely, GIPCs and GlcCers dominated this lipid group in the two *Ancylonema* species, and the same was reported also for leaves of land plants [60].

Generally, the knowledge of FA composition of conjugating algae is limited [61]. In a study on *Zygnema* [62], young and old cultures were compared. Young ones had only a low content of lipids and the most abundant FAs were palmitic acid, hexadecatrienoic and α-linolenic acid. During maturation, the most striking increase was found for oleic acid and linoleic acid, which rose by up to 17-fold and 8-fold, respectively [62]. The snapshot of fatty acid profiles between *A. nordenskioeldii* and *A. alaskana*, taken close to the end of the melting season at alpine glaciers (i.e., September), showed that the predominant fatty acids of both glacial blooms were palmitic acid, oleic acid, linoleic, α-linolenic and steriadonic acid. Similarly, these five FAs were the most abundant and accounted altogether for 93.8% of all fatty acids in the subantarctic soil alga *Fottea pyrenoidosa* SAG 1.88 [61]. In this work, almost half of the FAs were PUFAs, which are important to keep membrane fluidity at low temperatures [63]. However, the overall cellular content of FAs per dry weight was quite low.

## 5. Conclusions

This study demonstrated that *Ancylonema* can no longer be regarded as a monotypic genus for a filamentous glacial alga. Except from molecular traits, the main diagnostic feature of this genus are parietal hemispheric, cup-shaped or elongate-lobed chloroplasts while “true” *Mesotaenium* are characterized by an axial, plate-like chloroplast. Unicellular glacial alga *A. alaskana* (here revised from *M. berggrenii* var. *alaskanum*; previously referred to with an improper Latin termination of the infraspecific name as “*alaskana*“) is widespread in European glaciers, but it has to be tested how much species are actually behind the phenomenon in a global point of view. Physiologically, *A. nordenskioeldii* and *A. alaskana* are closely related organisms with similar or identical cellular lipid profiles and identical kind of phenolic secondary pigmentation. The photophysiologies of both species have to be compared with caution since the sampling sites were not the same. Nonetheless, the photosystems of *A. alaskana* were able to use efficiently low irradiances but showed higher ETR rates throughout the rapid light curve as compared to *A. nordenskioeldii*. Finally, the ecological reason why *A. alaskana* dominates communities at central European glaciers and, vice versa, *A. nordenskioeldii* dominates Arctic ones, remains open.

## Figures and Tables

**Table 1:** Glacial algal sample codes, collection date, sampling sites, elevation (in meters above sea level) and geographic position (GPS).

Sample	Harvest	Glacier	Elevation	GPS
P39	6 September 2006	Tiefenbach Ferner, AT	3000	46°55′ N 10°56′ E
WP167	30 August 2017	Gurgler Ferner, AT	2728	46°48.280′ N 10°58.804′ E
WP210	22 August 2018	Morteratsch Glacier, CH	2211	46°24.999′ N 9°56.021′ E
WP211	22 August 2018	Morteratsch Glacier, CH	2275	46°24.847′ N 9°56.032′ E
WP213	24 August 2018	Morteratsch Glacier, CH	2161	46°25.195′ N 9°55.913′ E
WP249	18 August 2020	Morteratsch Glacier, CH	2203	46°25.075′ N 9°55.985′ E
WP251	25 August 2020	Gurgler Ferner, AT	2727	46°48.327′ N 10°58.739′ E

**Table 2:** Average cell sizes (µm) ± standard deviation and cell size ranges (with n = number of measured cells in brackets), and maximum population densities ± standard deviation of the two glacial algae *A. alaskana* and *A. nordenskioeldii* are shown.

Species	Sample	Cell Width × Length (µm)	Cells mL^−1^
*A. alaskana*	P39 ^1^	8.7 ± 0.8 × 12.1 ± 2.8; 7.3–11.6 × 8.1–20.6 (83)	–
*A. alaskana*	WP167	7.6 ± 0.3 × 12.1 ± 1.9; 7–8.4 × 8.5–17.1 (30)	4.77 × 10^5^ ± 0.07 × 10^5^
*A. alaskana*	WP212	8.5 ± 0.7 × 12.8 ± 2.7; 7.5–10.2 × 9.4–19.7 (22)	–
*A. alaskana*	WP251	8.4 ± 0.8 × 11.4 ± 2.4; 7.0–10.5 × 8.4–16.3 (27)	–
*A. nordenskioeldii*	WP210	12.9 ± 1.1 × 29.4 ± 6.8; 10.7–15.2 × 19.6–50 (19)	–
*A. nordenskioeldii*	WP211	13.6 ± 0.8 × 37.5 ± 7.6; 12.4–14.7 × 22.1–51 (28)	4.8 × 10^5^ ± 0.25 × 10^5^
*A. nordenskioeldii*	WP249	13.7 ± 0.7 × 28.3 ± 5.5; 12.6–15 × 20.6–43.4 (33)	–

^1^ Published data are from [6].

## Data Availability

The obtained sequences (MW922838; MW922839; MW922840) were submitted in GenBank.

## Supplementary Materials

The following are available online at https://www.mdpi.com/article/10.3390/microorganisms9051103/s1, Figure S1: Overview of the sampling sites with blooms of glacial algae: (a) Gurgler Ferner, a glacier in Ötztal Valley, Tyrol, Austria (30 August 2017, sample WP167), (b) Morteratsch Glacier in Engadin, Graubünden, Switzerland (22 August 2018, sample WP211). Figure S2: Light micrographs of the Morteratsch Glacier field sample (WP249) showing (a) *Ancylonema alaskana* (small arrow; former *Mesotaenium berggrenii* var. *alaskanum*) and *Ancylonema nordenskioeldii* (large arrow). (b) Putative *Ancylonema nordenskioeldii* var. *chodatii* (arrowhead). Scale = 10 µm. Figure S3: Comparison of (a, c) cell widths and (b, d) cell lengths between *Ancylonema nordenskioeldii* (*A. n*.; WP210, n = 19; WP211, n = 28; WP249, n = 33) and *Ancylonema alaskana* (*A. a*.; P39, n = 83; WP167, n = 30; WP213, n = 22; WP251, n = 27). Details of sample locations are listed in Table 2. Figure S4: Light micrographs of aged field samples kept for a prolonged period under laboratorial conditions, showing (a-e) *Ancylonema alaskana* WP251 and (f, g) *Ancylonema nordenskioeldii* WP211. *Ancylonema alaskana* with gone vacuolar pigmentation: (a) prior division of the chloroplast and the pyrenoid (right cell); (b) just after the chloroplasts division; (c) arrowhead indicates the circle pyrenoid; (d) asterisks point to formation of the new wall (septum); (e) separation of the two young cells (i.e., a chain fragmentation), the fully developed septum (large arrow), single nucleus (n; behind the chloroplast). *Ancylonema nordenskioeldii* with altered vacuolar, ochre pigmentation: (f) Arrowhead indicates the circle pyrenoid. Actively dividing cells resulting in temporal presence of the two parietal partly lobed chloroplasts per each cell in filament, central translucent area shows the nucleus (*n*); (g) an old filament with a single chloroplast per cell and striking vacuolization. Scale: 10 µm. Figure S5: (a) high-performance liquid chromatography (HPLC)-chromatogram (at 280 nm) of a 20% ethanolic extract of *Ancylonema nordenskioeldii* field sample from Morteratsch Glacier, acquired with a diode array detector. Inserts: HPLC online spectra (b) second major peak (likely gallic acid glycoside) and (c) first major peak (likely purpurogallin carboxylic acid-6-O-β-D-glucopyranoside). Table S1: List of primers used for amplification of 18S rRNA gene (18S) and *rbc*L markers (F, forward; R, reverse). Table S2: List of 346 molecular species of lipids identified by shotgun analysis for *Ancylonema nordenskioeldii* (WP211) and *Ancylonema alaskana* (WP167). Table S3: List of glaciers in the Austrian Alps with occurrence of *Ancylonema alaskana*. No filaments of *Ancylonema nordenskioeldii* were found in all cases.
