# Supplementary material for: Unicellular versus Filamentous: The Glacial Alga Ancylonema alaskana comb. et stat. nov. and Its Ecophysiological Relatedness to Ancylonema nordenskioeldii (Zygnematophyceae, Streptophyta)
Source: Microorganisms. 2021 May 20;9(5):1103. doi: 10.3390/microorganisms9051103 (PMC8161032; doi:10.3390/microorganisms9051103)
Supplement: Supplementary file 1 [file microorganisms-09-01103-s001.zip › microorganisms-1214088-supplementary.pdf]

Supplementary Information

Unicellular versus Filamentous: the Glacial Alga *Ancylonema alaskana* comb. et stat. nov. and Its Ecophysiological Relatedness to *Ancylonema nordenskioeldii* (Zygnematophyceae, Streptophyta)

Lenka Procházková, Tomáš Řezanka, Linda Nedbalová and Daniel Remias

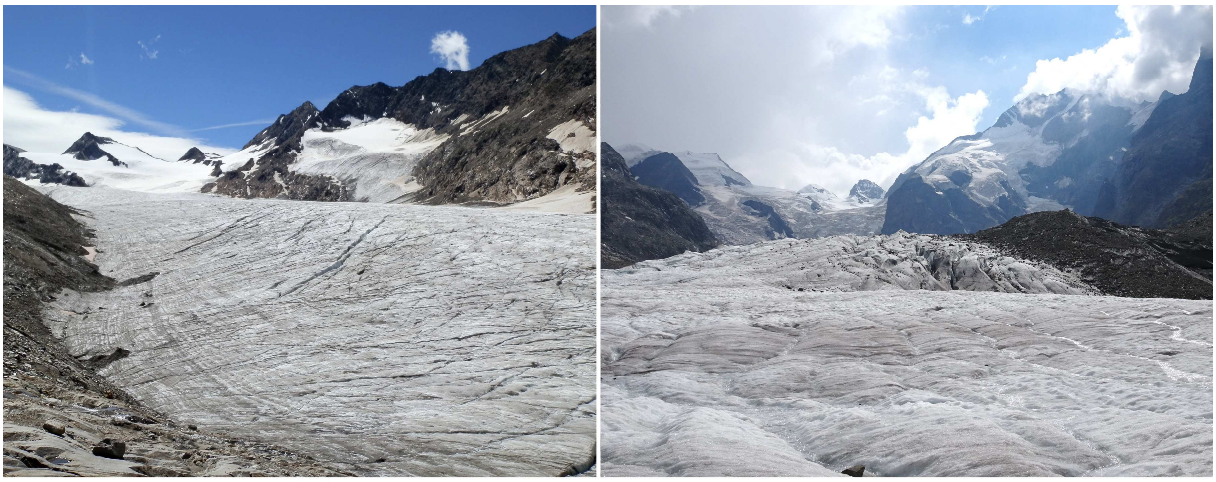

(a) (b)

Figure S1. Overview of the sampling sites with blooms of glacial algae: (**a**) Gurgler Ferner, a glacier in Ötztal Valley, Tyrol, Austria (30 Aug 2017, sample WP167), (**b**) Morteratsch Glacier in Engadin, Graubünden, Switzerland (22 Aug 2018, sample WP211).

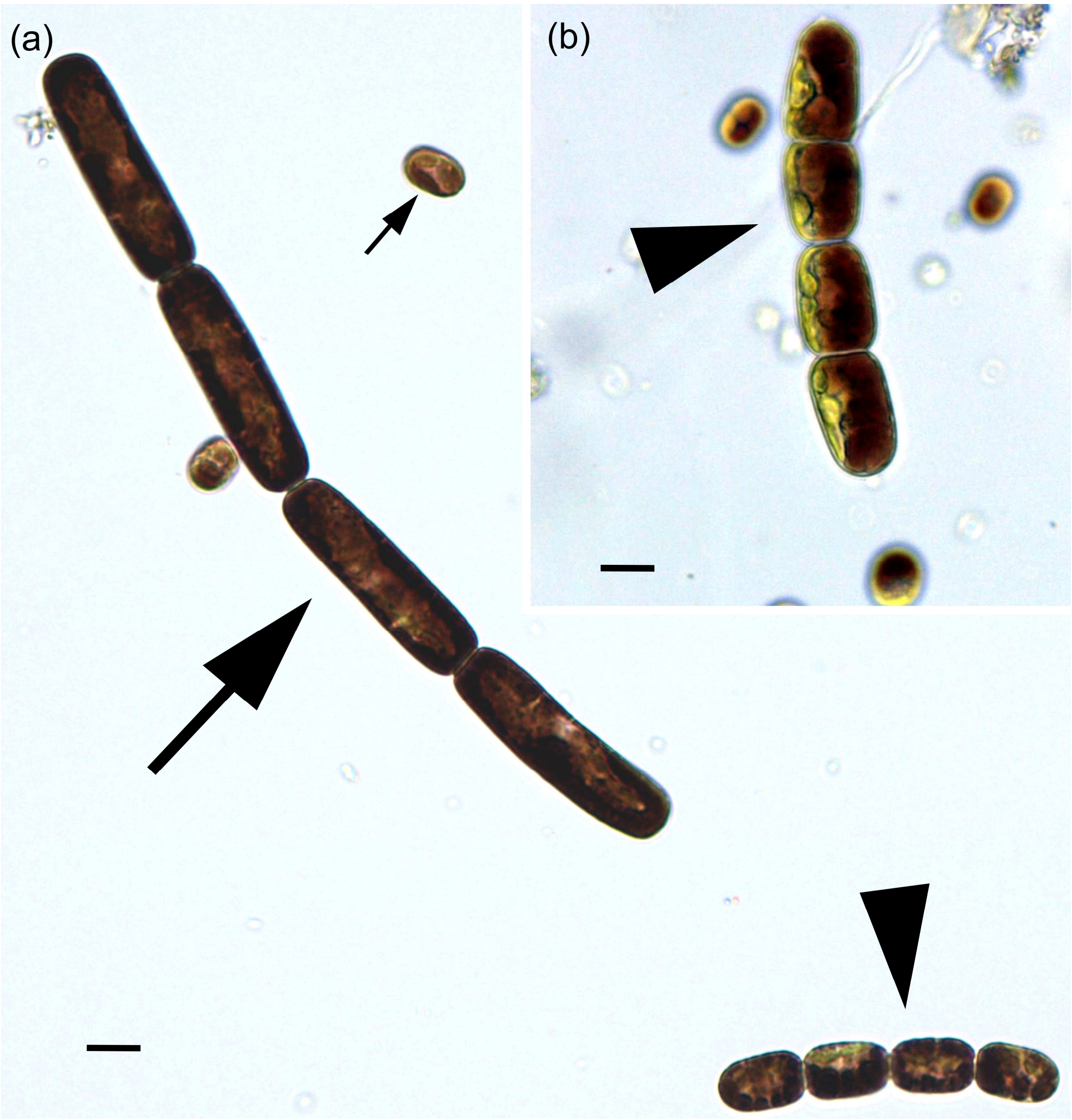

Figure S2. Light micrographs of the Morteratsch Glacier field sample (WP249) showing (**a**) *Ancylonema alaskana* (small arrow; former *Mesotaenium berggrenii* var. *alaskanum*) and *Ancylonema nordenskioeldii* (large arrow). (**b**) Putative *Ancylonema nordenskioeldii* var. *chodatii* (arrowhead). Scale = 10 μm.

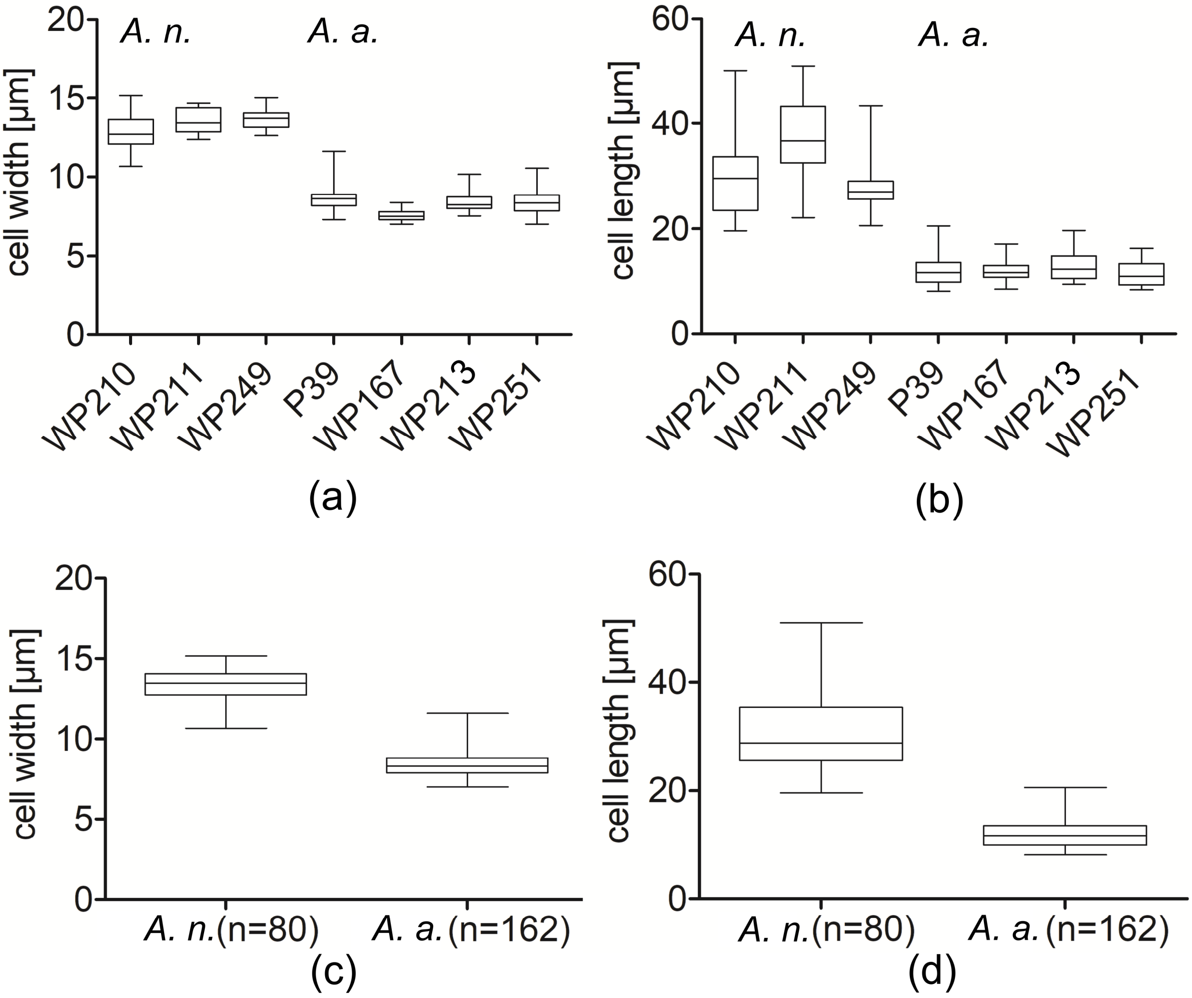

Figure S3. Comparison of (**a**, **c**) cell widths and (**b**, **d**) cell lengths between *Ancylonema nordenskioeldii* (*A. n.*; WP210, n=19; WP211, n=28; WP249, n=33) and *Ancylonema alaskana* (*A. a.*; P39, n=83; WP167, n=30; WP213, n=22; WP251, n=27). Details of sample locations are listed in Table 2.

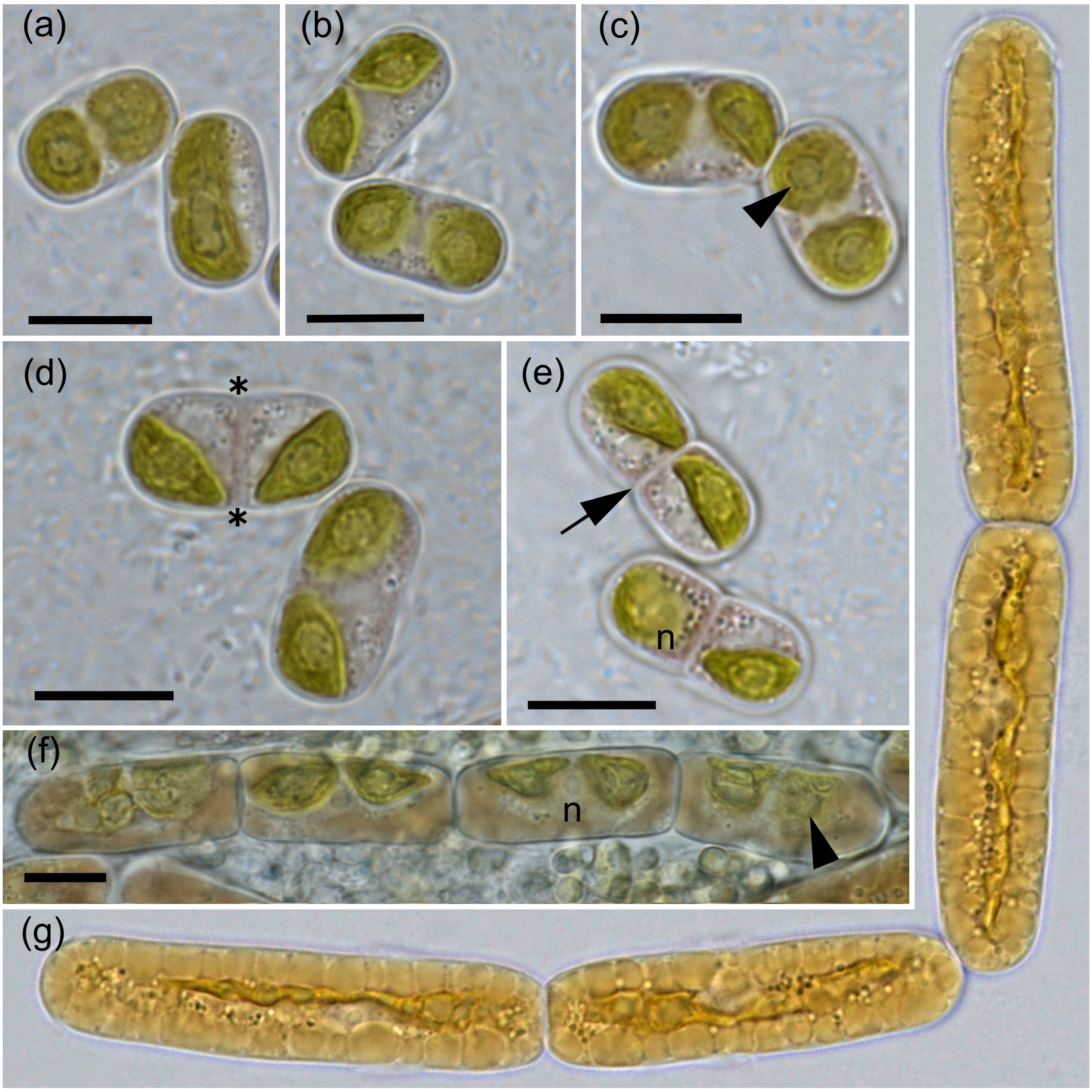

Figure S4. Light micrographs of aged field samples kept for a prolonged period under laboratorial conditions, showing (**a**-**e**) *Ancylonema alaskana* WP251 and (**f**, **g**) *Ancylonema nordenskioeldii* WP211. *Ancylonema alaskana* with gone vacuolar pigmentation: (**a**) prior division of the chloroplast and the pyrenoid (right cell); (**b**) just after the chloroplasts division; (**c**) arrowhead indicates the circle pyrenoid; (**d**) asterisks point to formation of the new wall (septum); (**e**) separation of the two young cells (i.e. a chain fragmentation), the fully developed septum (large arrow), single nucleus (n; behind the chloroplast). *Ancylonema nordenskioeldii* with altered vacuolar, ochre pigmentation: (**f**) Arrowhead indicates the circle pyrenoid. Actively dividing cells resulting in temporal presence of the two parietal partly lobed chloroplasts per each cell in filament, central translucent area shows the nucleus (*n*); (**g**) an old filament with a single chloroplast per cell and striking vacuolization. Scale: 10 μm.

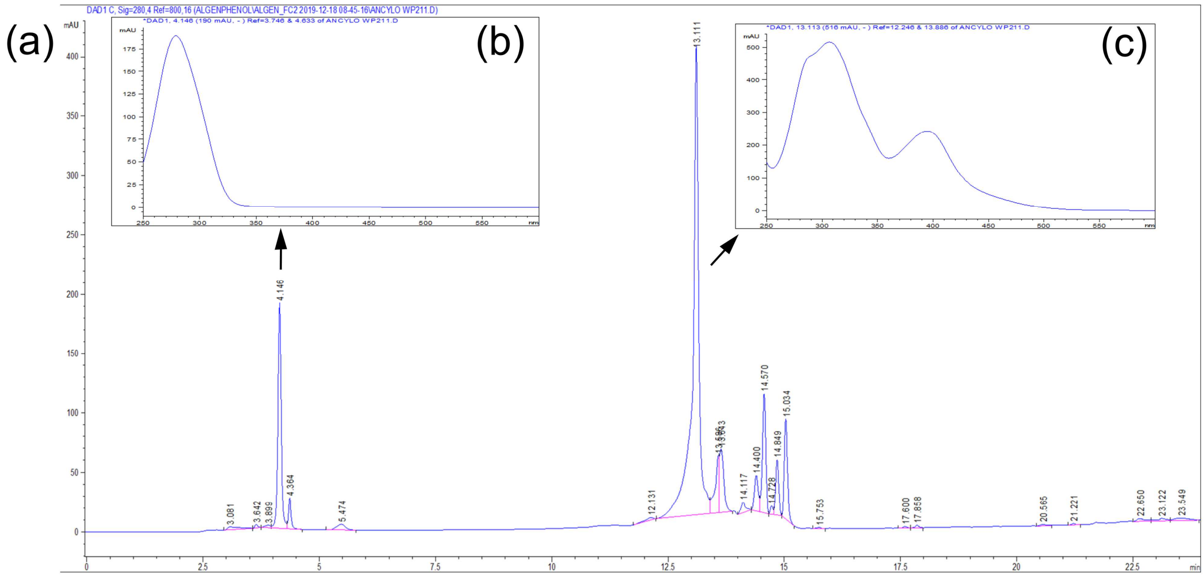

Figure S5. (**a**) HPLC-chromatogram (at 280 nm) of a 20% ethanolic extract of *Ancylonema nordenskioeldii* field sample from Morteratsch Glacier, acquired with a diode array detector. Inserts: HPLC online spectra (**b**) second major peak (likely gallic acid glycoside) and (**c**) first major peak (likely purpurogallin carboxylic acid-6-O-β-D-glucopyranoside).

Table S1. List of primers used for amplification of 18S rRNA gene (18S) and *rbc*L markers (F, forward; R, reverse).

Primer	Marker	Direction	Sequence	Reference
18F2	18S	F	AACCTGGTTGATCCTGCCAGT	[1]
18R2	18S	R	TGATCCTTCTGCAGGTTCACCTACG	[1]
P2	18S	F	CTGGTTGATTCTGCCAGT	[2]
P4	18S	R	TGATCCTTCYGCAGGTTCAC	[3]
MaGo1F	rbcL	F	ATGTCACCACAAACNGAAAC	[4]
rbcL7R	rbcL	R	AAATAAATACCACGGCTACG	[5]
rbcLKF2	rbcL	F	ACTTACTACACTCCTGATTATGA	[6]
R3	rbcL	R	ATRAAACGGTCTCTCCAACGCAT	[7]

References

1. Katana, A.; Kwiatowski, J.; Spalik, K.; Zakryś, B.; Szalacha, E.; Szymańska, H. Phylogenetic position of *Koliella* (Chlorophyta) as inferred from nuclear and chloroplast small subunit rDNA. *J. Phycol*. **2001**, 37, 443–51. https://doi.org/10.1046/j.1529-8817.2001.037003443.x
2. De Wever, A.; Leliaert, F.; Verleyen, E.; Vanormelingen, P.; Van der Gucht, K.; Hodgson, D.A.; Sabbe, K; Vyverman, W. Hidden levels of phylodiversity in Antarctic green algae: further evidence for the existence of glacial refugia. *P. Roy. Soc. B.-Biol. Sci*. **2009**, 276, 3591–9. DOI:10.1098/rspb.2009.0994.
3. Moon-van der Staay, S.Y.; van der Staay, G.W.M.; Guillou, L.; Vaulot, D.; Claustre, H.; Medlin, L.K. Abundance and diversity of prymnesiophytes in the picoplankton community from the equatorial Pacific Ocean inferred from 18S rDNA sequences. *Limnol. Oceanogr*. **2000**, 45, 98–109. https://doi.org/10.4319/lo.2000.45.1.0098
4. Gontcharov, A.A.; Marin, B.; Melkonian, M. Are Combined Analyses Better Than Single Gene Phylogenies? A Case Study Using SSU rDNA and rbcL Sequence Comparisons in the Zygnematophyceae (Streptophyta). *Mol. Biol. Evol*. **2004**, 21, 612–24. 10.1093/molbev/msh052
5. Hoham, R.W.; Tomas A. Bonome.; Christopher W. Martin.; James H. Leebens-Mack. A combined 18S rDNA and rbcL phylogenetic analysis of *Chloromonas* and *Chlamydomonas* (Chlorophyceae, Volvocales) emphasizing snow and other cold-temperature habitats. *J. Phycol*. **2002**, 38, 1051–64. DOI:10.1046/j.1529-8817.2002.t01-1-01227.x.
6. Škaloud, P.; Rindi, F. Ecological differentiation of cryptic species within an asexual protist morphospecies: A case study of filamentous green alga *Klebsormidium* (Streptophyta). *J. Eukar. Microbiol*. **2013**, 60, 350–62. DOI: 10.1111/jeu.12040
7. Matsuzaki, R.; Kawai-Toyooka, H.; Hara, Y.; Nozaki, H. Revisiting the taxonomic significance of aplanozygote morphologies of two cosmopolitan snow species of the genus *Chloromonas* (Volvocales, Chlorophyceae). *Phycologia*. **2015**, 54, 491–502. https://doi.org/10.2216/15-33.1

Table S2. List of 346 molecular species of lipids identified by shotgun analysis for *Ancylonema nordenskioeldii* (WP211) and *Ancylonema alaskana* (WP167).

**Compound Name**	***Ancylonema alaskana***	***Ancylonema nordenskioeldii***
Cers (d18:0, c16:0)	0.007	0.008
Cers (d18:1, c16:0)	0.006	0.006
Cers (t18:0, c16:0)	0.012	0.007
Cers (t18:0, c22:0)	0.003	0.003
Cers (t18:0, c24:0)	0.015	0.015
Cers (t18:0, c24:1)	0.003	0.003
Cers (t18:0, c26:0)	0.006	0.008
Cers (t18:0, c26:1)	0.001	0.001
Cers (t18:1, c16:0)	0.028	0.018
Cers (t18:1, c20:0)	0.001	0.001
Cers (t18:1, c22:0)	0.009	0.007
Cers (t18:1, c24:0)	0.039	0.033
Cers (t18:1, c24:1)	0.006	0.006
Cers (t18:1, c26:0)	0.027	0.03
Cers (t18:1, c26:1)	0.005	0.005
DAG (32:1)	0.154	0.126
DAG (32:2)	0.158	0.102
DAG (32:3)	0.135	0.064
DAG (32:4)	0.088	0.085
DAG (32:5)	0.092	0.104
DAG (32:6)	0.157	0.177
DAG (32:7)	0.036	0.048
DAG (32:8)	0.034	0.044
DAG (34:1)	0.029	0.034
DAG (34:2)	0.254	0.237
DAG (34:3)	0.372	0.372
DAG (34:4)	0.012	0.011
DAG (34:5)	0.006	0.007
DAG (34:6)	0.128	0.161
DAG (34:7)	0.061	0.098
DAG (34:8)	0.114	0.176
DAG (36:1)	0.004	0.003
DAG (36:2)	0.015	0.015
DAG (36:3)	0.017	0.018
DAG (36:4)	0.108	0.118
DAG (36:5)	0.354	0.403
DAG (36:6)	0.269	0.329
DAG (36:7)	0.041	0.104
DAG (36:8)	0.037	0.03
DAG (38:6)	0.07	0.113
DAG (38:7)	0.129	0.173
DAG (40:5)	0.14	0.179
DAG (40:8)	0.197	0.231

DGDG(34:1)	0.017	0.01
DGDG(34:2)	0.044	0.018
DGDG(34:3)	0.229	0.112
DGDG(34:4)	0.01	0.005
DGDG(34:5)	0.027	0.012
DGDG(34:6)	0.079	0.036
DGDG(36:1)	0.000	0.001
DGDG(36:2)	0.001	0.001
DGDG(36:3)	0.013	0.01
DGDG(36:4)	0.023	0.012
DGDG(36:5)	0.056	0.025
DGDG(36:6)	0.993	0.505
DGDG(38:3)	0.001	0.000
DGDG(38:4)	0.001	0.001
DGDG(38:5)	0.003	0.001
DGDG(38:6)	0.025	0.024
GIPCs (d18:0, h16:0)	0.072	0.104
GIPCs (d18:0, h18:0)	0.002	0.003
GIPCs (d18:0, h20:0)	0.009	0.01
GIPCs (d18:0, h22:0)	0.012	0.024
GIPCs (d18:0, h22:1)	0.003	0.000
GIPCs (d18:0, h24:0)	0.432	0.501
GIPCs (d18:0, h24:1)	0.094	0.086
GIPCs (d18:0, h26:0)	0.067	0.063
GIPCs (d18:1, h16:0)	0.266	0.172
GIPCs (d18:1, h18:0)	0.004	0.004
GIPCs (d18:1, h20:0)	0.099	0.05
GIPCs (d18:1, h22:0)	0.138	0.065
GIPCs (t18:0, h16:0)	0.059	0.076
GIPCs (t18:0, h18:0)	0.005	0.003
GIPCs (t18:0, h20:0)	0.013	0.018
GIPCs (t18:0, h22:0)	0.597	0.479
GIPCs (t18:0, h22:1)	0.03	0.005
GIPCs (t18:0, h24:0)	0.436	0.567
GIPCs (t18:0, h24:1)	0.339	0.389
GIPCs (t18:0, h26:0)	0.204	0.168
GIPCs (t18:1, h16:0)	0.317	0.185
GIPCs (t18:1, h18:0)	0.017	0.008
GIPCs (t18:1, h20:0)	0.116	0.063
GIPCs (t18:1, h20:1)	0.003	0.002
GIPCs (t18:1, h22:0)	1.805	1.382
GIPCs (t18:1, h22:1)	0.047	0.028
GIPCs (t18:1, h24:0)	4.629	3.958
GIPCs (t18:1, h24:1)	3.354	2.628
GIPCs (t18:1, h26:0)	1.678	1.356
GIPCs (t18:1, h26:1)	0.539	0.401
GlcCers (d18:0, h16:0)	0.007	0.007

GlcCers (d18:1, h16:0)	1.374	1.368
GlcCers (d18:1, h20:0)	0.005	0.000
GlcCers (d18:1, h22:0)	0.056	0.077
GlcCers (d18:1, h24:0)	0.122	0.261
GlcCers (d18:1, h24:1)	0.074	0.069
GlcCers (d18:1, h26:0)	0.029	0.057
GlcCers (d18:2, h16:0)	0.03	0.051
GlcCers (t18:0, h16:0)	0.011	0.019
GlcCers (t18:0, h18:0)	0.017	0.035
GlcCers (t18:0, h20:0)	0.003	0.008
GlcCers (t18:0, h22:0)	0.013	0.023
GlcCers (t18:0, h24:0)	0.005	0.041
GlcCers (t18:0, h24:1)	0.091	0.18
GlcCers (t18:0, h26:0)	0.01	0.026
GlcCers (t18:0, h26:1)	0.014	0.022
GlcCers (t18:1, h16:0)	0.384	0.47
GlcCers (t18:1, h18:0)	0.086	0.112
GlcCers (t18:1, h20:0)	0.091	0.103
GlcCers (t18:1, h20:1)	0.001	0.006
GlcCers (t18:1, h22:0)	0.774	1.210
GlcCers (t18:1, h22:1)	0.073	0.105
GlcCers (t18:1, h24:0)	1.864	3.707
GlcCers (t18:1, h24:1)	2.270	3.988
GlcCers (t18:1, h26:0)	0.831	1.455
GlcCers (t18:1, h26:1)	0.404	0.626
hCers (d18:0, h16:0)	0.055	0.1
hCers (d18:1, h16:0)	0.019	0.019
hCers (d18:1, h24:0)	0.001	0.001
hCers (d18:1, h26:0)	0.001	0.001
hCers (t18:0, h16:0)	0.007	0.015
hCers (t18:0, h22:0)	0.009	0.01
hCers (t18:0, h24:0)	0.032	0.037
hCers (t18:0, h24:1)	0.012	0.013
hCers (t18:0, h26:0)	0.012	0.017
hCers (t18:0, h26:1)	0.002	0.002
hCers (t18:1, h16:0)	0.091	0.068
hCers (t18:1, h18:0)	0.003	0.002
hCers (t18:1, h20:0)	0.006	0.004
hCers (t18:1, h22:0)	0.075	0.06
hCers (t18:1, h22:1)	0.003	0.003
hCers (t18:1, h24:0)	0.215	0.22
hCers (t18:1, h24:1)	0.123	0.111
hCers (t18:1, h26:0)	0.113	0.108
hCers (t18:1, h26:1)	0.028	0.024
LCBs (d18:0)	0.032	0.018
LCBs (d18:1)	0.000	0.003
LCBs (t18:0)	0.091	0.146

LCBs (t18:0-P)	0.001	0.001
LCBs (t18:1)	0.031	0.053
LCBs (t18:1-P)	0.001	0.001
LysoPC(16:0)	0.001	0.001
LysoPC(18:2)	0.002	0.002
LysoPC(18:3)	0.001	0.001
LysoPE(16:0)	0.001	0.001
LysoPE(18:2)	0.002	0.002
LysoPE(18:3)	0.001	0.001
LysoPG(16:0)	0.001	0.000
LysoPG(16:1)	0.003	0.000
LysoPG(18:1)	0.001	0.001
LysoPG(18:2)	0.001	0.000
LysoPG(18:3)	0.008	0.004
MGDG(34:1)	0.013	0.012
MGDG(34:2)	0.031	0.012
MGDG(34:3)	0.118	0.073
MGDG(34:4)	0.161	0.078
MGDG(34:5)	0.956	0.459
MGDG(34:6)	5.567	2.850
MGDG(36:1)	0.002	0.000
MGDG(36:2)	0.002	0.000
MGDG(36:3)	0.006	0.004
MGDG(36:4)	0.055	0.028
MGDG(36:5)	0.178	0.076
MGDG(36:6)	1.754	1.006
MGDG(38:4)	0.001	0.000
MGDG(38:5)	0.003	0.002
MGDG(38:6)	0.007	0.007
PA(34:1)	0.000	0.002
PA(34:2)	0.006	0.012
PA(34:3)	0.005	0.009
PA(34:4)	0.000	0.001
PA(36:2)	0.001	0.002
PA(36:3)	0.001	0.002
PA(36:4)	0.005	0.009
PA(36:5)	0.003	0.008
PA(36:6)	0.001	0.002
PC(34:1)	0.047	0.023
PC(34:2)	0.441	0.255
PC(34:3)	0.361	0.253
PC(34:4)	0.014	0.008
PC(36:1)	0.005	0.003
PC(36:2)	0.062	0.047
PC(36:3)	0.138	0.09
PC(36:4)	0.44	0.229
PC(36:5)	0.503	0.295

PC(36:6)	0.218	0.137
PC(38:2)	0.007	0.004
PC(38:3)	0.006	0.004
PC(38:4)	0.008	0.004
PC(38:5)	0.005	0.003
PC(38:6)	0.002	0.001
PC(40:2)	0.002	0.001
PC(40:3)	0.005	0.002
PC(40:4)	0.005	0.003
PE(32:1)	0.002	0.001
PE(32:2)	0.003	0.002
PE(32:3)	0.002	0.001
PE(34:1)	0.008	0.005
PE(34:2)	0.437	0.219
PE(34:3)	0.255	0.168
PE(34:4)	0.003	0.003
PE(36:1)	0.001	0.000
PE(36:2)	0.029	0.023
PE(36:3)	0.057	0.04
PE(36:4)	0.218	0.152
PE(36:5)	0.211	0.145
PE(36:6)	0.061	0.043
PE(38:3)	0.004	0.003
PE(38:4)	0.004	0.002
PE(38:5)	0.003	0.002
PE(38:6)	0.003	0.002
PE(40:2)	0.01	0.008
PE(40:3)	0.002	0.003
PE(42:2)	0.009	0.012
PE(42:3)	0.008	0.011
PE(42:4)	0.002	0.003
PG(32:0)	0.064	0.061
PG(32:1)	0.125	0.089
PG(34:0)	0.014	0.016
PG(34:1)	0.158	0.121
PG(34:2)	0.296	0.19
PG(34:3)	0.558	0.443
PG(34:4)	1.040	0.566
PG(36:1)	0.002	0.001
PG(36:2)	0.003	0.002
PG(36:3)	0.004	0.006
PG(36:4)	0.004	0.003
PG(36:5)	0.005	0.005
PG(36:6)	0.006	0.003
PI(32:0)	0.000	0.005
PI(32:1)	0.005	0.007
PI(32:2)	0.002	0.004

PI(32:3)	0.007	0.01
PI(34:1)	0.004	0.004
PI(34:2)	0.207	0.154
PI(34:3)	0.189	0.165
PI(34:4)	0.004	0.005
PI(36:2)	0.011	0.007
PI(36:3)	0.013	0.008
PI(36:4)	0.009	0.006
PI(36:5)	0.012	0.009
PI(36:6)	0.014	0.007
PS(34:2)	0.022	0.009
PS(34:3)	0.022	0.01
PS(36:2)	0.008	0.004
PS(36:3)	0.009	0.006
PS(36:4)	0.003	0.002
PS(36:5)	0.003	0.001
PS(36:6)	0.001	0.001
PS(38:2)	0.01	0.006
PS(38:3)	0.01	0.005
PS(38:4)	0.001	0.001
PS(38:6)	0.001	0.000
PS(40:2)	0.013	0.01
PS(40:3)	0.011	0.008
PS(42:2)	0.02	0.02
PS(42:3)	0.023	0.022
PS(42:4)	0.005	0.005
PS(44:2)	0.001	0.001
PS(44:3)	0.000	0.001
SQDG (32:0)	2.412	2.463
SQDG (34:1)	0.455	0.456
SQDG (34:2)	2.517	2.384
SQDG (34:3)	1.903	2.595
TAG (42:0)	0.136	0.059
TAG (44:0)	0.154	0.063
TAG (44:1)	0.19	0.082
TAG (44:2)	0.09	0.034
TAG (46:0)	0.181	0.092
TAG (46:1)	0.28	0.112
TAG (46:2)	0.127	0.097
TAG (46:3)	0.109	0.073
TAG (46:4)	0.271	0.02
TAG (48:0)	1.286	1.279
TAG (48:1)	0.369	0.331
TAG (48:10)	0.233	0.387
TAG (48:11)	0.201	0.276
TAG (48:12)	0.276	0.381
TAG (48:2)	0.247	0.228

TAG (48:3)	0.841	0.562
TAG (48:4)	0.425	0.499
TAG (48:5)	0.355	0.446
TAG (48:6)	0.353	0.399
TAG (48:7)	0.275	0.32
TAG (48:8)	0.19	0.337
TAG (50:0)	0.168	0.147
TAG (50:1)	0.446	0.41
TAG (50:10)	0.283	0.243
TAG (50:11)	0.235	0.16
TAG (50:12)	0.208	0.122
TAG (50:2)	0.353	0.314
TAG (50:3)	2.276	2.502
TAG (50:4)	3.180	3.471
TAG (50:5)	3.753	4.017
TAG (50:6)	0.454	0.617
TAG (50:7)	4.176	4.809
TAG (50:8)	0.371	0.345
TAG (50:9)	0.497	0.532
TAG (52:1)	0.095	0.102
TAG (52:10)	0.444	0.495
TAG (52:11)	0.28	0.426
TAG (52:12)	0.262	0.321
TAG (52:2)	0.67	0.643
TAG (52:3)	0.766	0.73
TAG (52:4)	0.897	0.83
TAG (52:5)	2.467	2.465
TAG (52:6)	2.440	2.411
TAG (52:7)	0.554	0.384
TAG (52:8)	1.919	2.105
TAG (52:9)	0.463	0.403
TAG (54:1)	0.452	0.218
TAG (54:10)	0.285	0.345
TAG (54:11)	0.222	0.314
TAG (54:12)	0.181	0.32
TAG (54:2)	0.062	0.228
TAG (54:3)	1.058	0.737
TAG (54:4)	0.572	0.427
TAG (54:5)	0.535	0.437
TAG (54:6)	0.631	0.822
TAG (54:7)	1.866	2.437
TAG (54:8)	3.605	4.635
TAG (54:9)	2.918	3.848
TAG (56:0)	0.072	0.061
TAG (56:1)	0.049	0.047
TAG (56:10)	0.039	0.069
TAG (56:11)	0.025	0.05

TAG (56:12)	0.039	0.079
TAG (56:13)	0.024	0.04
TAG (56:3)	0.06	0.049
TAG (56:4)	0.045	0.037
TAG (56:5)	0.049	0.011
TAG (56:6)	0.016	0.041
TAG (56:7)	0.024	0.033
TAG (56:8)	0.035	0.044
TAG (56:9)	0.022	0.04
TAG (58:1)	0.042	0.033
TAG (58:10)	0.023	0.044
TAG (58:11)	0.018	0.032
TAG (58:12)	0.013	0.023
TAG (58:13)	0.018	0.037
TAG (58:14)	0.026	0.045
TAG (58:2)	0.028	0.024
TAG (58:4)	0.02	0.015
TAG (60:12)	0.025	0.043
TAG (60:13)	0.026	0.056
TAG (60:14)	0.022	0.044
TAG (60:15)	0.013	0.023

Table S3. List of glaciers in the Austrian Alps with occurrence of *Ancylonema alaskana*. No filaments of *Ancylonema nordenskioeldii* were found in all cases.

Glacier name	Land
Gurgler Ferner	Tyrol, Austria
Rettenbachferner	Tyrol, Austria
Tiefenbach Ferner	Tyrol, Austria
Daunkogelferner	Tyrol, Austria
Vernagtferner	Tyrol, Austria
Winnebach Ferner	Tyrol, Austria
Rotmoosferner	Tyrol, Austria
Langtaler Ferner	Tyrol, Austria
Pasterze	Carinthia, Austria
